# Serum keratan sulfate transiently increases in the early stage of osteoarthritis during strenuous running of rats: protective effect of intraarticular hyaluronan injection

**DOI:** 10.1186/ar2363

**Published:** 2008-01-30

**Authors:** Tao Tang, Takeshi Muneta, Young-Jin Ju, Akimoto Nimura, Kyosuke Miyazaki, Hiroyuki Masuda, Tomoyuki Mochizuki, Ichiro Sekiya

**Affiliations:** 1 Section of Orthopedic Surgery, Graduate School, Tokyo Medical and Dental University, 1-5-45 Yushima, Bunkyo-ku, Tokyo 113-8519, Japan; 2 Center of Excellence Program of Frontier Research on Molecular Destruction and Reconstruction of Tooth and Bone, Tokyo Medical and Dental University, 1-5-45 Yushima, Bunkyo-ku, Tokyo 113-8519, Japan; 3 Department of Pharmaceuticals Information, Seikagaku Corporation, 1-6-1 Marunouchi, Chiyoda-ku, Tokyo 113-8519, Japan; 4 Section of Cartilage Regeneration, Graduate School, Tokyo Medical and Dental University, 1-5-45 Yushima, Bunkyo-ku, Tokyo 113-8519, Japan

## Abstract

**Introduction:**

Osteoarthritis is influenced by genetic and environment factors, including mechanical stress; however, the relationship between running and the development of osteoarthritis remains a matter of controversy. We investigated whether osteoarthritic change could be obtained in a rat strenuous running model, whether serum keratan sulfate in rats could be detected by HPLC and was associated with onset or progression of osteoarthritis, and whether hyaluronan injection suppressed development of osteoarthritis and elevation of serum keratan sulfate.

**Methods:**

Wistar rats were forced to run 30 km in 6 weeks on a treadmill machine. Articular cartilage of the knees was evaluated macroscopically and immunohistologically. Serum keratan sulfate was examined every week by HPLC. The effect of weekly knee injection of hyaluronan was also investigated.

**Results:**

Cartilage surfaces stained with India ink became irregular, metachromasia by safranin-O staining appeared to be almost lost, and Mankin's score significantly worsened after 30 km of running. Serum keratan sulfate in rats was detected by HPLC and transiently increased (peaked at 3 to 4 weeks) along with depletion of keratan sulfate in cartilage tissue. Hyaluronan treatment suppressed morphological progression of osteoarthritis and elevation of serum keratan sulfate.

**Conclusion:**

Rat strenuous running induced osteoarthritis. Serum keratan sulfate was associated with progression of osteoarthritis. Weekly intraarticular injection of hyaluronan controlled the development of osteoarthritis, and the effect was reflected by serum keratan sulfate.

## Introduction

Osteoarthritis is the most common cause of joint pain and loss of mobility in older people. Osteoarthritis is influenced by genetic and environmental factors, including mechanical stress. To overcome difficulties in studying osteoarthritis in humans, animal models have been developed, such as spontaneous models in aging animals, genetically modified mice, as well as surgically, enzymatically or chemically induced models [1,2]. The use of strenuous running helps simulate long-term stress on weight-bearing joints. This model does not require surgical procedures or injection of reagents, and therefore it can detect subtle symptoms of osteoarthritis. The relationship between running and the development of osteoarthritis, however, remains a matter of controversy [3-7]. The first purpose of our study was to examine whether osteoarthritic change could be obtained in a rat strenuous running model.

Keratan sulfate is a glycosaminoglycan that is specifically distributed in the extracellular matrix of the cartilage, cornea, and brain [8]. Serum keratan sulfate was measured using an ELISA in 1985, and its usefulness as a marker of osteoarthritis was proposed; however, serum keratan sulfate did not correlate with the X-ray grading [9,10]. In 2007 Wakitani and colleagues measured serum keratan sulfate using HPLC, which has been reported to be more sensitive and more accurate than ELISA [11], and demonstrated a higher value of serum keratan sulfate in patients with early-stage damage of the articular cartilage undetectable by X-ray imaging [12]. These results indicate a more important usefulness of HPLC for serum keratan sulfate. On the other hand, recent analysis for serum keratan sulfate in rats and mice has scarcely been investigated possibly due to one paper describing the absence of keratan sulfate in skeletal tissues of mouse and rat [13]. In this paper, keratan sulfate was examined by immunohistochemistry using the monoclonal antibody MZ15; however, keratan sulfate expression in rat cartilage was detected using the other antibodies 5D4 [14] and EFG-11 [6]. The second study purpose was to investigate serum keratan sulfate in rats by HPLC and its association with onset or progression of osteoarthritis.

Hyaluronan is also a glycosaminoglycan. In articular cartilage, hyaluronan and aggrecan form large aggregates, bind huge amounts of water, and are responsible for the resilience of cartilage. Intraarticular injection of hyaluronan has been wildly utilized clinically as pain relief for the early stage of knee osteoarthritis [15,16]. Several studies have reported that hyaluronan has beneficial effects on cartilage during development of osteoarthritis [17]. The third study objective was to analyze serum keratan sulfate sequentially after hyaluronan injection in a rat strenuous running model.

## Materials and methods

### Animals and strenuous running

Wistar rats 16 to 18 weeks of age (Sankyo Labo Service, Tokyo, Japan) were used for the experiments. All experiments were conducted in accordance with the institutional guidelines for the care and use of experimental animals. Rats were divided into three groups: no running group (0 km, *n *= 5); only strenuous running group (30 km, *n *= 8); and strenuous running and hyaluronan injection group (15 km, *n *= 3; 30 km, *n *= 5).

**Figure 1:**
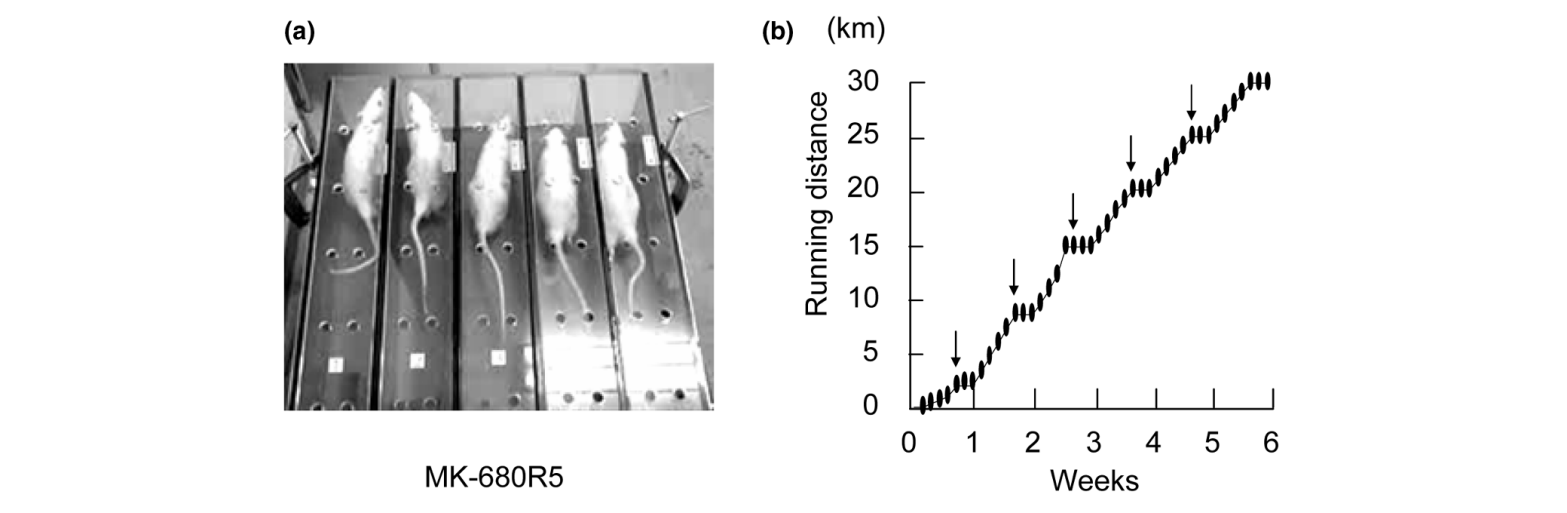
Method for rat strenuous running. **(a) **Rodent treadmill machine designed to compulsively make rats run. **(b) **Protocol for running exercise. Rats were forced to run 30 km in 6 weeks. For the hyaluronan group, the injection was performed initially at 5 days, followed by every 7 days (arrows).

For strenuous running exercise, a rodent treadmill machine (MK-680R5; ME Service., Tokyo, Japan) was used with a 5% incline (Figure 1a). The MK-680R5 has been designed to compulsively make animals exercise by electrical shock delivered to the animals without failure by the adoption of a shock generator scrambler. The rats were acclimated to the treadmill by gradually increasing the running speed and time as follows: day 1, 10 minutes at 10 m/min; day 2, 15 minutes at 12 m/min; day 3, 20 minutes at 15 m/min; day 4, 30 minutes at 18 m/min; and day 5, 35 minutes at 20 m/min. On day 8 and thereafter, the rats were forced to run for 55 minutes a day at 20 m/min, with the first 10 minutes consisting of a 12 m/min warm-up. Rats ran 30 km in 6 weeks, as shown in Figure 1b[18]. For the hyaluronan injection group (*n *= 5), 100 μl hyaluronan (average molecular weight = 8 × 10^5 ^Da; Seikagaku Corp., Tokyo, Japan) containing 1 mg in formulated concentrate was injected into the right knee. The injections were performed initially at 5 days and every 1 week thereafter under anesthesia of 10 mg sodium pentobarbital (Dainippon Sumitomo Pharma, Osaka, Japan) by intraperitoneal injections. As a control for hyaluronan treatment, saline or PBS was not injected into the left knee to avoid the possibility that they might enhance osteoarthritis [19,20].

### Macroscopic observation

Femoral and tibial condyles were carefully dissected separately without damaging the cartilage surface, and were then stained with India ink to identify the location, size and severity of cartilage degeneration. Macroscopic pictures were taken using a specification MPS-7 (Sugiura Laboratory Inc., Tokyo, Japan), a dedicated medical photography platform. Digital images were taken using a Nikon Coolpix 4500 digital camera (Nikon, Tokyo, Japan).

### Histology

The rats were sacrificed with an overdose of sodium pentobarbital. Both femurs and tibias were fixed in 4% paraformaldehyde at pH 7.4 for 3 days, were decalcified in 20% ethylenediamine tetraacetic acid solution for 21 days, and were then embedded in paraffin wax. Femurs and tibias were sectioned sagittally at 5 μm and stained with safranin-O. Histological sections were visualized using an Olympus IX71 microscope (Olympus, Tokyo, Japan). Each section was evaluated with the Mankin's histological grading system (Mankin's score: 0 to 14) for articular cartilage degeneration [21].

### Immunohistochemistry

Sections were deparaffinized, washed in PBS, and pretreated with 0.4 mg/ml proteinase K (DAKO, Carpinteria, CA, USA) in Tris–HCl buffer for 15 minutes at room temperature for optimal antigen retrieval. Endogenous peroxidases were quenched using 0.3% hydrogen peroxide in methanol for 20 minutes at room temperature. The sections were rinsed once in PBS for 5 minutes and were briefly blocked with 10% normal horse serum (Vector Laboratories, Burlingame, CA, USA) to avoid nonspecific binding of the antibody. The sections were then incubated in monoclonal anti-keratan sulfate antibody (5-D-4, 1:100 dilution with PBS containing 1% BSA; Seikagaku Corp.) or anti-mouse monoclonal antibody against human type II collagen (1:200 dilution with PBS containing 1% BSA; Daiichi Fine Chemical, Toyama, Japan) at room temperature for 60 minutes. After rinsing in PBS, the tissues were incubated with biotinylated horse anti-mouse IgG secondary antibody (Vector Laboratories) for 30 minutes at room temperature. The slides were again immersed in PBS and were incubated for another 30 minutes with Vectastain ABC reagent (Vector Laboratories). Finally, the sections were shortly counterstained with hematoxylin, dehydrated and mounted in a xylol-soluble mount (Vitro-Clud; R. Langenbrinck, Emmendingen, Germany) [12,22,23].

### Keratan sulfate concentration

To avoid circadian variation of keratan sulfate in serum, blood was collected at between 3:00 and 4:00 pm, 1 hour after finishing strenuous running. Approximately 500 μl blood was aspirated with a 27-gauge needle from the tail vein of the rats. The blood was kept at 4°C for 2 hours and was centrifuged at 2,000 rpm for 15 minutes at 4°C. The serum was separated, allocated into 100 μl, and kept frozen at -70°C. To avoid an influence of freeze–thaw, the serum was used for the analyses without refreezing. Every rat started strenuous running on Monday and had its blood aspirated every Friday. Each 200 μl serum was diluted with 800 μl distilled water, digested with 100 μl of 2.0% Actinase E (Kaken Pharmaceutical., Tokyo, Japan) at 55°C for 24 hours, and was heated at 100°C for 10 minutes. The whole solution was applied to Q Sepharose 0.15 M sodium chloride (GE Healthcare UK Ltd., Little Chalfont, Buckinghamshire, UK), and was extracted with 50 mM Tris–HCl buffer (pH 8.6) containing 2 M sodium chloride. The extracted material was desalinated with PD-10 (GE Healthcare), dried, and dissolved again by 0.2 ml distilled water. Then 1 mU Keratanase II (Seikagaku Corp.) was added, followed by addition of 40 μl of 100 mM sodium acetate buffer (pH 6.0), and the mixture was incubated at 37°C for 3 hours.

The sample was ultrafiltered using an Ultrafree C3GC system whose molecular weight cutoff was 10,000 daltons (Japan Millipore, Tokyo, Japan). The filtrate, which contained monosulfate disaccharide and disulfate disaccharide derived from keratan sulfate, was analyzed by HPLC with the column packed with polyamine-bound silica (YMC gel PA-120; YMC Ltd, Kyoto, Japan). The monosulfate disaccharide and disulfate disaccharide were eluted with a gradient of 0 to 100 mM sodium sulfate for 45 minutes at a flow rate of 0.5 ml/min. To elute from the column, 100 mM sodium tetraborate buffer (pH 9.0) containing 1% 2-cyanoacetamide was added at a flow rate of 0.5 ml/min. The mixture was passed through poly(ether ether ketone) (PEEK; Victrex, Lancashire, UK) tubing with a 0.5 mm diameter and a 10 m length in a dry fluoromonitor (excitation, 331 nm; emission, 383 nm). The area of each peak corresponding to monosulfate disaccharide and to disulfate disaccharide was calculated by Borwin-HSS2000 software (Jasco, IJsselstein, Netherlands) and was converted to the amount of the corresponding disaccharides against the area of standard monosulfate disaccharide and disulfate disaccharide (Seikagaku Corp.) [12].

### Statistical analysis

The StatView 5.0 program (SAS Institute, Cary, NC, USA) was used for statistical analyses. *P *< 0.05 was considered statistically significant.

## Results

### Degeneration of articular cartilage

**Figure 2:**
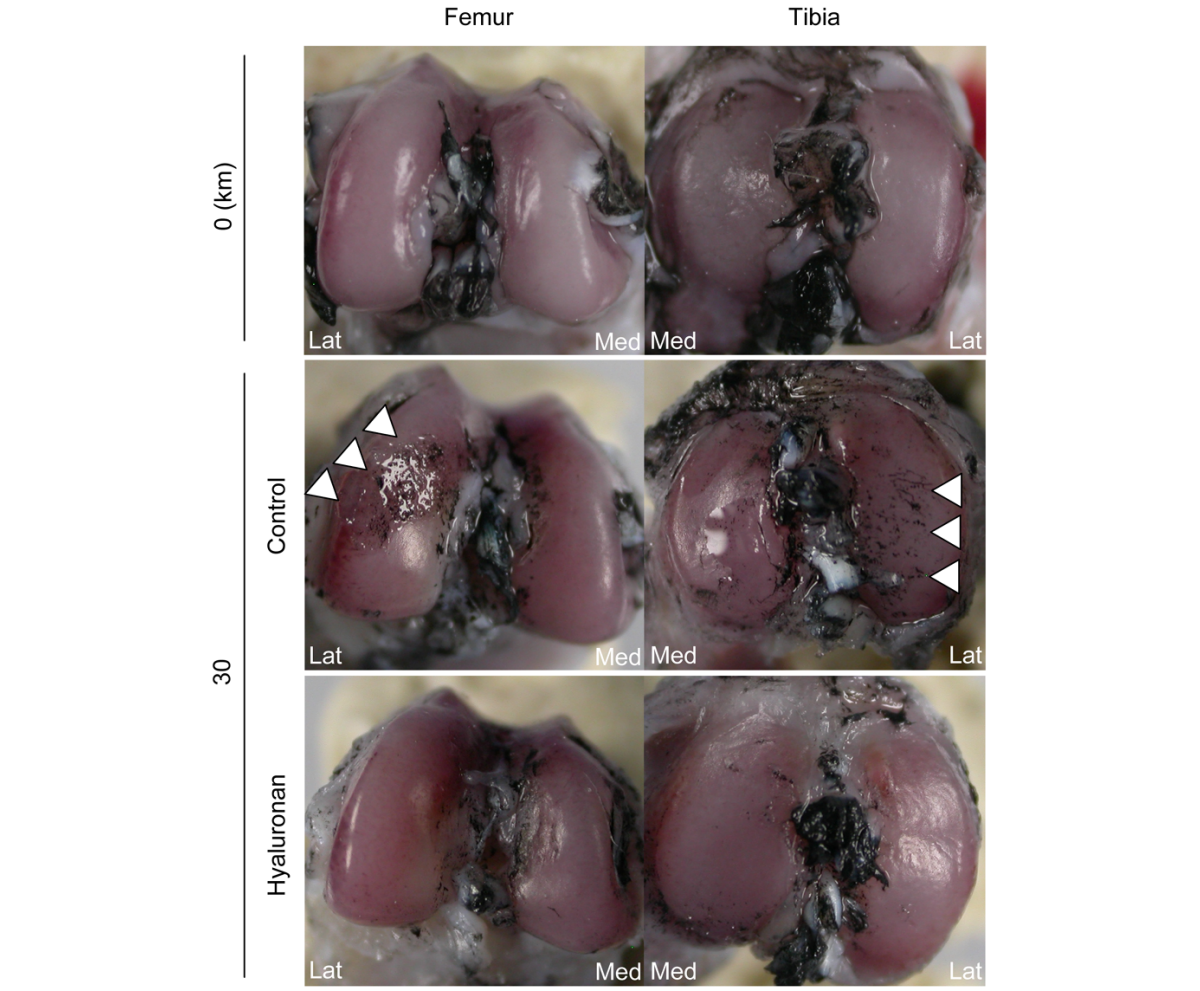
Macroscopic observation. Femoral and tibial articular cartilage stained with India ink. Cartilage lesions are indicated by arrowheads. Lat, lateral; Med, medial.

**Figure 3:**
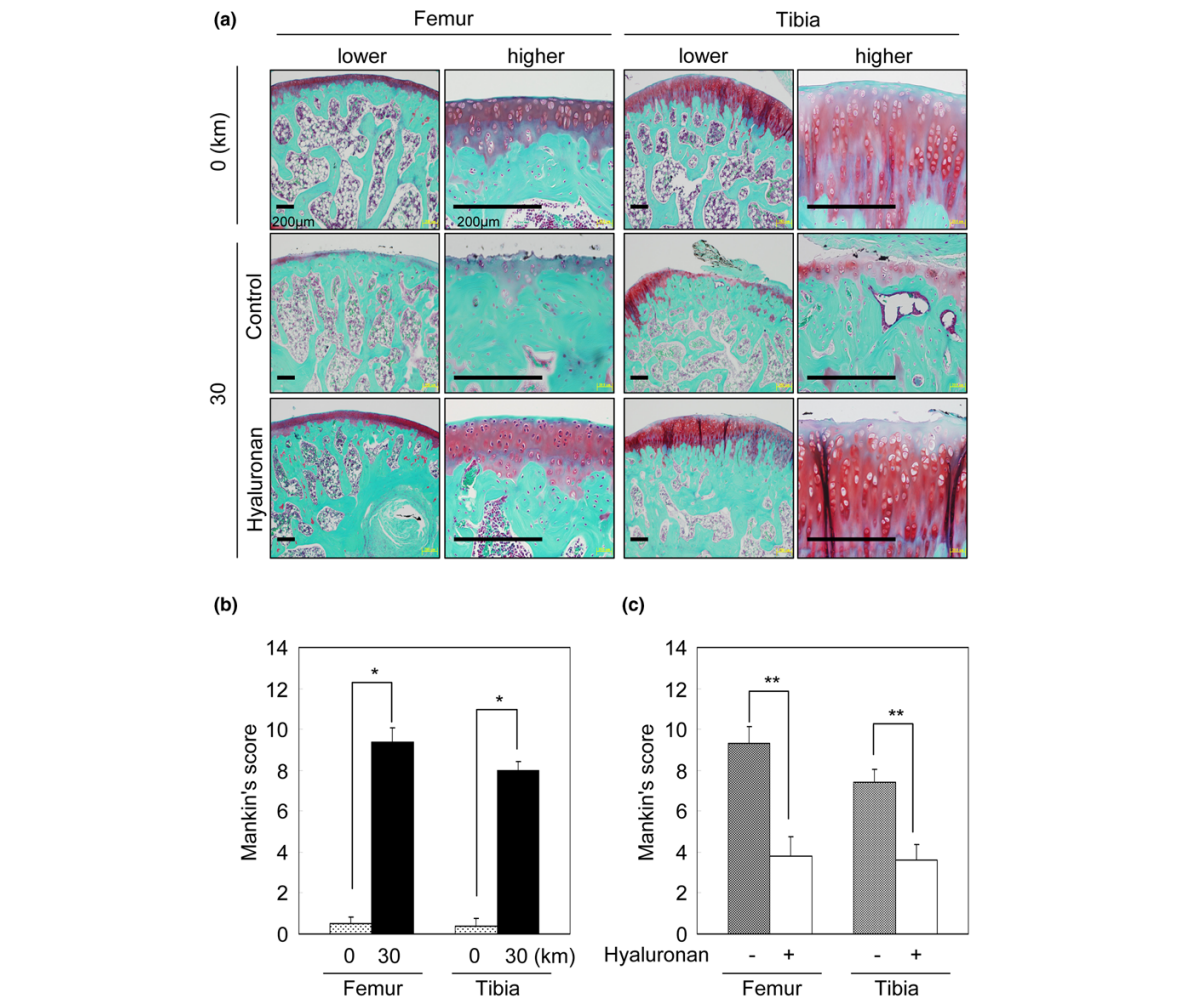
Histological analyses for cartilage legions. **(a) **Representative histologies of the lateral femoral condyle and the lateral tibial plateau stained with safranin-O. Scale bar = 200 μm. **(b) **Influence of running 30 km on cartilage. Mankin's score of the right knee in rats after 30 km of strenuous running without hyaluronan injection was compared with that in control rats without strenuous running or hyaluronan injection. Data expressed as the mean ± standard deviation (*n *= 5). *P < 0.01, Mann–Whitney U test. **(c) **Effect of hyaluronan on cartilage. Mankin's score for cartilage legions. Rats were forced to run 30 km in 6 weeks. Hyaluronan was injected each week into the right knee. Nothing was injected into the left knee. Both sides of the knees were compared. Mankin's score expressed as the mean ± standard deviation (*n *= 5). **P < 0.05, Wilcoxon signed rank test.

Strenuous running exercise affected the articular cartilage. The cartilage surfaces of both the lateral femoral condyle and the lateral tibial plateau were irregular after 30 km of strenuous running (Figure 2). A weekly intraarticular hyaluronan injection helped maintain the smoothness of the surface of the articular cartilage (Figure 2). Histological analyses demonstrated that 30 km of strenuous running induced depletion of the articular cartilage matrix (Figure 3a) and worsened the Mankin's score (Figure 3b). Hyaluronan treatment suppressed the degeneration of the articular cartilage (Figure 3c).

### Serum concentration of keratan sulfate

**Figure 4:**
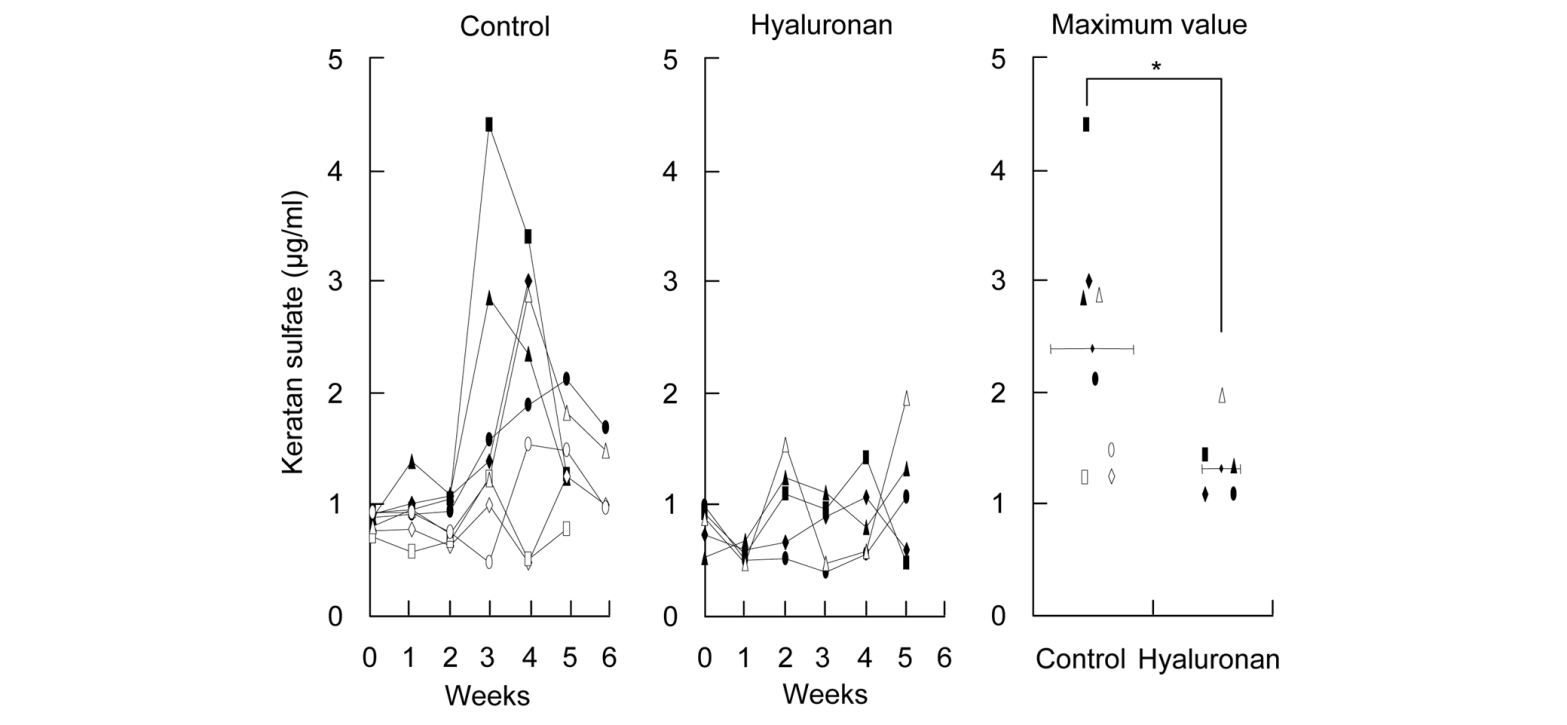
Serum concentration of keratan sulfate. Blood was collected every week at 0 to 5 weeks. Sequential serum concentrations of keratan sulfate are shown individually in the control group (left panel) and in the hyaluronan group (center panel). Maximum values of the concentrations are plotted, and the average values are shown in the right panel (control, *n *= 8; injection of hyaluronan, *n *= 5). **P < 0.05 by Mann–Whitney U test.

Sequential serum concentrations of keratan sulfate were examined. In the control group (the only strenuous running group), the concentration transiently peaked at 3 to 4 weeks (Figure 4, left). Hyaluronan treatment appeared to suppress the keratan sulfate concentration (Figure 4, center). The average of the maximum keratan sulfate concentration during the 5-week period in each rat in the hyaluronan group was significantly lower than that in the control group (Figure 4, right).

### Immunohistochemical analysis

**Figure 5:**
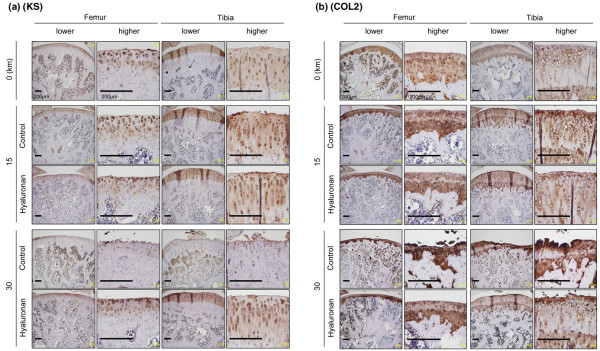
Immunohistochemical analyses. Representative histologies of the lateral femoral condyle and the lateral tibial plateau immunostained with **(a) **keratan sulfate (KS) and **(b) **type II collagen (COL2). Scale bar = 200 μm.

Keratan sulfate and type II collagen were expressed in cartilage of rats before strenuous running and were still present at 3 weeks after 15 km of strenuous running. Keratan sulfate expression decreased and was hardly observed at 6 weeks after 30 km of strenuous running in the control group. The type II collagen-positive area decreased along with the cartilage area, but the expression could be still observed in the remaining cartilage in the control group after 30 km of strenuous running. Hyaluronan treatment suppressed the loss of keratan sulfate and type II collagen after 30 km of strenuous running (Figure 5).

## Discussion

Running exercise may injure articular cartilage – although there are also studies suggesting that it has no adverse effects on articular cartilage, and that the effects are mostly beneficial [5]. These studies indicate that the influences of physical exercise are bidirectional, the net result dependent on the degree of joint loading. Excessive running seems to lead to a higher incidence of osteoarthritis, whereas moderate running is either noncontributory in joint degeneration or beneficial in decreasing the risk of osteoarthritis in animals. Excessive running load is expected to markedly exceed the animals' normal physiological running activities. Pap and colleagues first reported development of osteoarthritis in the knee joints of rats after strenuous running exercise [3]. They stimulated the rats intracranially to motivate them to run on a running wheel. In our present study, we used a rodent treadmill machine to motivate Wistar rats running by external electrical stimulation to their tail. We demonstrated development of osteoarthritis of the knee joint of nongenetically modified rats. This simple method without requiring specific surgery will be useful for analyses of subtle symptoms such as serum cartilage marker in the present study.

Progression of osteoarthritis is likely to result primarily from an imbalance between cartilage degradation and repair. Biological markers in the blood might provide relevant information more rapidly than imaging techniques such as radiography and magnetic resonance imaging can, and should contribute to our understanding of mechanisms that underlie the clinical efficacy of osteoarthritis treatments [24]. As osteoarthritis affects mainly the cartilage, structural molecules or fragments derived from cartilage tissues could be candidate serum cartilage markers for osteoarthritis. These tissues might also include molecules that play a role in metabolic processes, such as cytokines, proteases, and enzyme inhibitors. In human subjects, serum keratan sulfate increased after exercise in healthy athletes [25] and in patients with early-stage osteoarthritis [12]. Furthermore, keratan sulfate was specifically distributed in the cartilage, cornea, and brain [8]. We therefore focused on serum keratan sulfate as an osteoarthritis marker in this rat model.

In the present study we demonstrated that serum keratan sulfate rapidly increased when Wistar rats ran approximately 15 to 20 km. Interestingly, serum keratan sulfate rapidly decreased thereafter. According to our immunohistochemical analysis, keratan sulfate expression was still stable when rats ran 15 km and disappeared when rats ran 15 to 20 km. We speculate a possible mechanism as follows. Daily strenuous exercise was extremely difficult for rats, and much of the resulting mechanical stress was absorbed by their joints. This caused transient joint cartilage degradation. Proteoglycan fragments, which are detached by mechanical stress to the cartilage, were ejected from the synovial cavity into the blood through the lymphatic system, and consequently serum keratan sulfate increased. Keratan sulfate in the affected cartilage also rapidly disappeared after keratan sulfate degraded once, and then serum keratan sulfate decreased rapidly.

Uebelhart and colleagues previously reported that serum keratan sulfate increased sharply 1 day after injection of chymopapain into the knee joint in rabbits [26]. The serum keratan sulfate also sharply decreased, and these changes were accompanied by depletion of proteoglycans evaluated by safranin-O stained histology. Although the cartilage matrix degradated in a much shorter period and keratan sulfate expression was not analyzed spatially or temporally in their model, their results correspond with our results in that serum keratan sulfate levels increased predictably following acute loss of proteoglycan.

For evaluation of keratan sulfate, we digested rat serum with Keratanase II and then measured the sum of monosulfate and disulfate disaccharides derived from keratan sulfate by HPLC. Rat serum may contain nonsulfate disaccharides, whose level may be affected by degeneration of the articular cartilage. In the present study, we cannot answer how the error of ignoring nonsulfated disaccharides could affect the measurement of the total keratan sulfate concentration in rat serum. We could, however, demonstrate that sulfated disaccharides derived from keratan sulfate in rat serum transiently increased in the early stages of osteoarthritis.

Our immunohistological analysis has shown that strenuous running led to damage of type II collagen in 6 weeks. We examined sequentially the serum concentration of C2C, specific for the destruction of type II collagen by MMP-1, MMP-8, and MMP-13. We could not, however, detect C2C in rat serum in 6 weeks (data not shown).

Previous *in vitro *and *in vivo *studies indicate that exogenous hyaluronan can enhance proteoglycan synthesis and can prevent its release from the cell matrix [16,27]. Hyaluronan also suppresses the production and activity of proinflammatory mediators and proteases as well as altering the function of immune cells [17]. Intraarticular hyaluronan injection can reduce painful symptoms and improve general activities and joint mobility [28]. The mechanism may be that intraarticular hyaluronan injection causes less friction between articular cartilages, and improves joint comeback and reduces pain, thus providing good balance of the knee joint for running exercise.

Lammi and colleagues investigated the distribution of endogenous hyaluronan in full thickness defects of rat articular cartilage [6]. In normal articular cartilage, hyaluronan was stained mainly around the chondrocytes. During repair, strong hyaluronan staining was observed in loose mesenchymal tissue and in an area undergoing endochondral ossification. The high level of endogenous hyaluronan, however, could not induce the repair of osteochondral defect. Interestingly, remarkably strong staining for hyaluronan was demonstrated in areas that were simultaneously devoid of staining for keratan sulfate [6]. These results may show the possibility that the effect of endogenous hyaluronan is insufficient to repair the cartilage defect, losing keratan sulfate expression.

The present *in vivo *study demonstrated that intraarticular injection of hyaluronan suppressed progression of osteoarthritis. One of the mechanisms was to prevent release of keratan sulfate from the cartilage matrix. This could be monitored by the concentration of keratan sulfate in serum.

## Conclusion

Osteoarthritic change could be obtained in a rat strenuous running model. Rat serum keratan sulfate was detected by HPLC and transiently increased along with depletion of keratan sulfate in cartilage tissue. Hyaluronan treatment suppressed development of osteoarthritis, and the effect was reflected by serum keratan sulfate.

## Abbreviations

BSA = bovine serum albumin; ELISA = enzyme-linked immunosorbent assay; HPLC = high-performance liquid chromatography; PBS = phosphate-buffered saline.

## Competing interests

The present work was supported by Seikagaku Corporation.

## Authors' contributions

TT carried out the animal experiments, analyzed the results, and drafted the manuscript. TMu designed the initial plan. Y-JJ, AN, and TMo assisted in the animal experiments. KM and HM examined the keratan sulfate concentration. IS conducted the experiments, participated in the evaluation, and completed the final manuscript. All authors read and approved the final manuscript.
